# Strong optical response and light emission from a monolayer molecular crystal

**DOI:** 10.1038/s41467-019-13581-9

**Published:** 2019-12-06

**Authors:** Huijuan Zhao, Yingbo Zhao, Yinxuan Song, Ming Zhou, Wei Lv, Liu Tao, Yuzhang Feng, Biying Song, Yue Ma, Junqing Zhang, Jun Xiao, Ying Wang, Der-Hsien Lien, Matin Amani, Hyungjin Kim, Xiaoqing Chen, Zhangting Wu, Zhenhua Ni, Peng Wang, Yi Shi, Haibo Ma, Xiang Zhang, Jian-Bin Xu, Alessandro Troisi, Ali Javey, Xinran Wang

**Affiliations:** 1 National Laboratory of Solid State Microstructures, School of Electronic Science and Engineering, and Collaborative Innovation Center of Advanced Microstructures, Nanjing University, Nanjing, 210093 China; 2 Department of Electrical Engineering and Computer Sciences, University of California at Berkeley, Materials Sciences Division, Lawrence Berkeley National Laboratory, Berkeley, CA 94720 USA; 3 Key Laboratory of Mesoscopic Chemistry of MOE, School of Chemistry and Chemical Engineering, Nanjing University, Nanjing, 210093 China; 4 Department of Electrical and Computer Engineering, University of Wisconsin, Madison, Madison, 53705 USA; 5 National Laboratory of Solid State Microstructures, Jiangsu Key Laboratory of Artificial Functional Materials, College of Engineering and Applied Sciences and Collaborative Innovation Center of Advanced Microstructures, Nanjing University, Nanjing, 210093 China; 6 NSF Nanoscale Science and Engineering Center (NSEC), University of California, Berkeley, CA 94720 USA; 7 School of Microelectronics, Xidian University, Xian, 710071 China; 8 Department of Physics, Southeast University, Nanjing, 211189 China; 9 Department of Electronic Engineering, The Chinese University of Hong Kong, Hong Kong, 999077 China; 10 Department of Chemistry, University of Liverpool, Liverpool, L69 7ZD U.K.

**Keywords:** Organic-inorganic nanostructures, Two-dimensional materials, Molecular self-assembly

## Abstract

Excitons in two-dimensional (2D) materials are tightly bound and exhibit rich physics. So far, the optical excitations in 2D semiconductors are dominated by Wannier-Mott excitons, but molecular systems can host Frenkel excitons (FE) with unique properties. Here, we report a strong optical response in a class of monolayer molecular J-aggregates. The exciton exhibits giant oscillator strength and absorption (over 30% for monolayer) at resonance, as well as photoluminescence quantum yield in the range of 60–100%. We observe evidence of superradiance (including increased oscillator strength, bathochromic shift, reduced linewidth and lifetime) at room-temperature and more progressively towards low temperature. These unique properties only exist in monolayer owing to the large unscreened dipole interactions and suppression of charge-transfer processes. Finally, we demonstrate light-emitting devices with the monolayer J-aggregate. The intrinsic device speed could be beyond 30 GHz, which is promising for next-generation ultrafast on-chip optical communications.

## Abstract (web summary)

The optical response of inorganic two-dimensional semiconductors is dominated by Wannier-Mott excitons, but molecular systems can host localised Frenkel excitons. Here, the authors report strong optical response in a class of monolayer molecular J-aggregates due to the coherent Coulomb interaction between localised Frenkel excitons.

## Introduction

The reduced Coulomb screening at low dimensions has led to many fascinating phenomena in two-dimensional (2D) materials^1^. The exciton binding energy can exceed hundreds of meV, more than one order of magnitude larger than bulk semiconductors and quantum-well structures^2,3^. In addition, the large charge-dipole and dipole-dipole interaction (~tens of meV) can stabilize many-body complexes such as trions and bi-excitons even at room temperature^4,5^. While most studies have focused on atomic crystals, many molecular semiconductors also have 2D layered form^6–10^. Compared to popular 2D semiconductors such as transition metal dichalcogenides (TMDs), the dipole-dipole interaction is even stronger in molecular systems given the localized nature of excitation (e.g., Frenkel excitons (FE) is localized on a single molecule) and low dielectric constant. Therefore, it is expected that dimensionality control of molecular semiconductors would substantially modify the excitonic coupling and lead to new optoelectronic functions.

A particularly interesting system to study exciton interaction is molecular J-aggregate. First observed in 1930s^11^, it is characterized by red-shift and narrowing of spectral lines due to constructive quantum superposition of monomer excitations^12^. These delocalized states are less perturbed by disorders (analogy of Bloch wave in crystalline solids) and therefore highly desirable for improving device functions^13^. However, in most cases the extent of coherence is rather limited because the energy relaxation is dominated by inter-molecular CT (also called self-trapping) in ultrafast sub-picosecond time scale, among other processes^14^. To this end, 2D assembly is extremely appealing due to enhanced Coulomb interaction between local Frenkel dipoles and complete suppression of interlayer CT. In fact, the most efficient light-harvesting complexes found in nature, the chlorosome, contains dye molecules organized into 2D lamellar form^15^.

Recently, we have developed vapor-transport-based means to precisely assembled molecules on 2D surfaces, opening the possibility to investigate their electrical and optical properties^16,17^. In this work, we study the optical properties of monolayer (ML) perylene derivatives, namely dimethyl-3,4,9,10-perylenetetracarboxilic diimide (Me-PTCDI), 3,4,9,10-perylene-tetracarboxylic diimide (PTCDI) and 3,4,9,10-perylene-tetracarboxylic dianhydride (PTCDA), where the molecular packing ensures strong dipole interaction but weak electronic coupling, an ideal condition for long-range J-aggregation. We find that the exciton has giant oscillator strength, leading to strong absorption (over 15 and 30% at room temperature and 4 K, respectively) and bright emission (more than two orders of magnitude brighter than semiconducting TMDs). Combined temperature-dependent experiments and density functional theory (DFT) calculations suggest that the size of exciton wavefunction (*N*_c_) gradually builds up and could reach the order of hundred molecules at low temperature, which is similar to Wannier excitons in inorganic semiconductors. We further fabricate transient light-emitting devices on ML perylene derivatives and show that the excitonic state is robust under electrical excitation. The observed near-100% photoluminescence quantum yield (PLQY), sub-30 ps radiative lifetime and electrical injection pave the way for high efficiency, high speed light-emitting devices enabled by coherent dipole interactions.

## Characterization and room-temperature optical properties of ML Me-PTCDI crystals

**Fig. 1:**
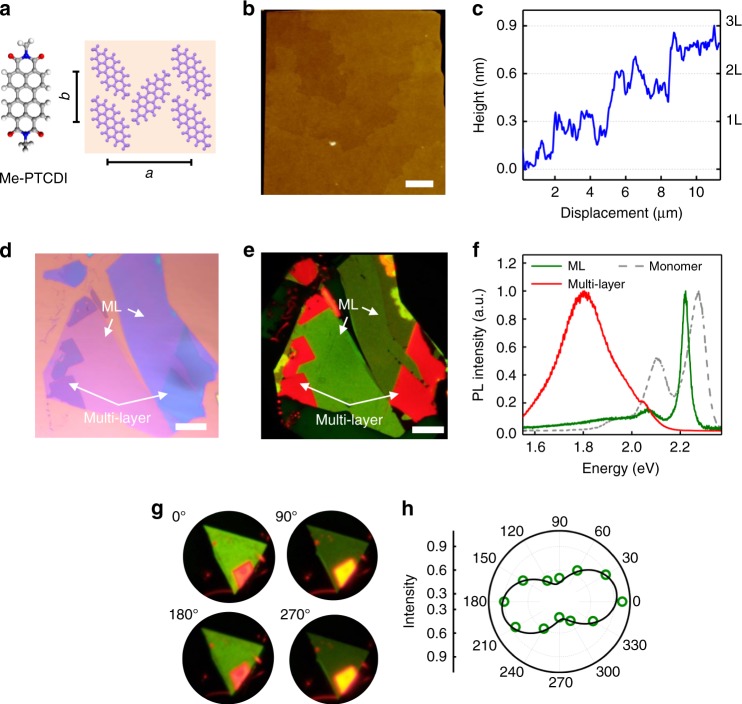
Structural characterization and thickness-dependent optical properties of 2D Me-PTCDI crystals. **a** In-plane molecular packing of ML Me-PTCDI on h-BN. **b** AFM image of a multi-layer Me-PTCDI sample showing the layered morphology. Scale bar: 2 μm. **c** The height profile of Me-PTCDI steps measured from (**b**). **d**, **e** Optical microscope and PL image of a Me-PTCDI sample with both ML and multi-layer regions marked by the white arrows. The different green colors in ML regions is due to slight variations of the spectrum (such as intensity ratio between the peaks). Scale bars: 10 μm. **f** PL spectra of ML Me-PTCDI, multi-layer and monomer at room temperature. **g** Polarization-resolved PL images of a ML Me-PTCDI samples at four different rotation angles. The uniform color change proves single crystalline nature of the ML. **h** Normalized PL intensity collected from ML region in (**g**).

To grow macroscopically high-quality perylene derivatives, we carried out physical vapor transport in a tube furnace using mechanically exfoliated hexagonal boron nitride (h-BN) as substrate^18^ (see “Methods” section). Layered morphology of all three molecular crystals with step height of ~3.3 Å down to ML was revealed by atomic force microscopy (AFM) (Fig. 1b, c, Supplementary Fig. 1), indicating face-on molecular packing. We did not observe any evidence of polymorphism between ML and multi-layer samples. Figure 1a shows the in-plane herringbone lattice structure of Me-PTCDI (*a* = 21.8 Å, *b* = 13.6 Å) determined from selected-area electron diffraction (SAED) and confirmed by high-resolution AFM (Supplementary Fig. 2). Our structural characterizations were consistent with previous experiments and molecular dynamics simulations on 2D surfaces^19,20^, but with slightly different lattice parameters from bulk (102) plane^21^ likely due to the interaction with h-BN substrate. Since the three molecules show qualitatively the same behavior, below we focus our discussion on Me-PTCDI (see Supplementary Note 1 for the discussion on PTCDA and PTCDI).

**Fig. 2:**
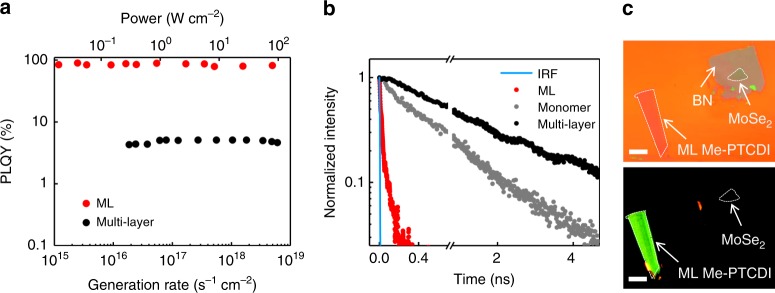
Thickness-dependent PLQY and lifetime at room temperature. **a** PLQY of a ML and multi-layer Me-PTCDI as a function of pump power (optical generation rate). **b** PL lifetime of Me-PTCDI ML, multi-layer and spin cast monomer, measured with streak camera (the instrument response function (IRF) is shown as reference). The ML data can be fitted with two components. The faster component (*τ* ~ 27 ps) is attributed to radiative recombination because of the high PLQY. The slower component (*τ* ~ 200 ps) could be non-radiative due to traps or disorder, which only contributes to 7.5% of the total excitation. The multi-layer and monomer data can be fitted with a single component with *τ* ~ 1.73 ns and 556 ps, respectively. **c** Room-temperature optical microscope (top) and PL image (bottom, under 450 nm excitation) of a ML Me-PTCDI and ML MoSe_2_ sample, both on h-BN. While the Me-PTCDI shows bright luminescence, the MoSe_2_ is nearly invisible. Scale bars: 10 μm.

The lack of *π*-*π* stacking in ML results in minimal in-plane electronic coupling and CT (Supplementary Fig. 3, Supplementary Table 1). On the other hand, reduced screening gives strong attractive dipole-dipole coupling *J* ~−23 meV and forms J-aggregation. The large *J* means that many-body interaction should be significant even at room temperature, leading to interesting layer-dependent optical properties. Figure 1e shows the PL image of a Me-PTCDI sample with both ML and multi-layer regions, taken by a color CCD camera under 450 nm LED illumination (Fig. 1d is the microscope image of the sample). While the multi-layer regions showed bulk-like red luminescence, the ML regions showed distinct green luminescence (this phenomenon was reproduced in all samples). The change of color was not gradual but occurred abruptly at ML (Supplementary Fig. 4). Furthermore, the PL was anisotropic with 180° period as shown by polarization-resolved PL imaging (Fig. 1g, h). The uniform change of intensity across the whole ML region suggested that it was single crystalline and that the green luminescence was an intrinsic property of ML crystal rather than from defects or impurities. The change of luminescent color was also reflected in PL spectrum, where ML and multi-layer samples peaked near 2.22 and 1.80 eV, respectively (Fig. 1f). We also compared the static and time-resolved PL with monomers spin cast on h-BN from very dilute dimethyl sulfoxide solution. We observed simultaneous red shift from monomer 0-0 Frenkel transition (by ~50 meV, Fig. 1f), reduced linewidth and vibronic progressions, as well as lifetime shortening by more than one order of magnitude (27 ps vs. 556 ps, Fig. 2), which were typical signs of superradiance in J-aggregate^12^. These experimental evidences suggested exciton delocalization over *N*_c_ ~10 molecules at room temperature, as expected from the large *J* and further corroborated by DFT calculations (Supplementary Fig. 10).

**Fig. 3:**
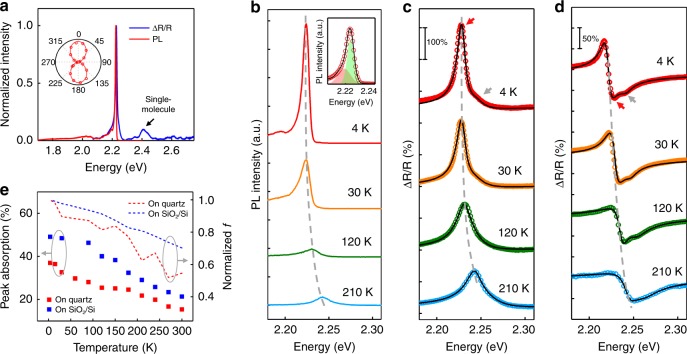
Temperature-dependent absorption and PL of ML Me-PTCDI. **a** Normalized PL and differential reflectance spectrum of a ML Me-PTCDI sample at 4 K. Inset: Polarization-dependent PL intensity. **b** Close-up temperature-dependent PL spectra of ML Me-PTCDI near the main peak. The gray dashed line shows the bathochromic shift of PL peak. **c**, **d** Close-up temperature-dependent differential reflectance spectra of ML Me-PTCDI on quartz (**c**) and SiO_2_/Si (**d**). The red and gray arrows represent the differential reflectance from superradient state and single-molecule FE, respectively. **e** Temperature-dependent absorption (squares) and normalized oscillator strength (dashed lines) of ML Me-PTCDI on quartz and SiO_2_/Si substrates, respectively, derived from transfer matrix method.

We measured the room temperature PLQY of ML Me-PTCDI under 2.41 eV excitation^22^, where the absorption was ~2.4 ± 0.2% (see “Methods” section). Remarkably, a significant portion of samples showed near-100% PLQY (Fig. 2a, see Supplementary Fig. 5b for statistics of 19 samples). As reference, the PLQY of multi-layer Me-PTCDI was only ~4.8% (Fig. 2a), due to the dominant inter-layer CT and H-aggregation effects^23,24^. The low PLQY for multi-layer samples is consistent with thin films^23^. Although single-molecule Me-PTCDI in solution can exhibit 93% quantum efficiency^23^, the near-100% PLQY in solid-state aggregate is unprecedented. Importantly, the PLQY of ML was maintained throughout the entire range of pump power without any decay, indicating that higher order non-radiative decay processes such as Auger and biexitonic recombination did not play important roles as in TMDs^22^. This is a huge advantage in device applications. Currently, even direct-gap TMDs and black phosphorous have low PLQY on the order of 10% or less^22,25,26^, which posts fundamental limit on the efficiency of light-emitting devices^3,27^. Although chemical treatment can improve the PLQY of metal-sulfides to near unity, it rolls off below 1% at high excitation density that is most relevant to devices^22,26^. To demonstrate the bright PL from ML Me-PTCDI, we directly compared with several TMDs (MoS_2_, WS_2_, MoSe_2_, WSe_2_) under 450 nm and 532 nm excitations. In Fig. 2c, while we observed bright luminescence from Me-PTCDI, the MoSe_2_ was nearly invisible. Quantitatively, the PL of ML Me-PTCDI was 2–5 orders of magnitude brighter than these TMDs under the same experimental conditions (Supplementary Fig. 6). We note that under non-resonant 450 nm and 532 nm excitations, the absorption of ML Me-PTCDI is lower than the TMDs^26^ (Fig. 3). Therefore, the much brighter PL from ML Me-PTCDI is due to enhanced PLQY.

## Temperature-dependent optical properties

To further study the exciton properties in ML perylene derivatives, we performed temperature-dependent optical measurements. Figure 3a shows the PL and differential reflectance (ΔR/R) spectrum of ML Me-PTCDI at 4 K (the same sample, on quartz substrate). The PL showed drastically reduced linewidth of 8.5 meV, little vibronic progression and nearly perfect linear polarization $\left( {P = \frac{{I_{{\mathrm{max}}} - I_{{\mathrm{min}}}}}{{I_{{\mathrm{max}}} + I_{{\mathrm{min}}}}} = 94.1{\mathrm{\% }}} \right)$, due to increased *N*_c_ at low temperature. The PL could be fitted with two Gaussians (separated by 5.2 meV, attributed to Davydov doublet from the two molecules in the unit cell^28^, Fig. 3b inset), indicating that the linewidth was inhomogeneously broadened due to the large number of molecules under laser spot. Interestingly, the absorption was also dominated by the same peak without Stokes shift (Fig. 3a). This means that the exciton gains very large oscillators strength such that it also dominates the absorption process. Aside from the main resonance peak, single-molecule FE also contributed to a small part of absorption as shown by the small peak on the high-energy shoulder (gray arrows in Fig. 3c, d) and its vibronic peak near 2.4 eV (arrow in Fig. 3a, Supplementary Fig. 7).

Quantitative absorption and oscillator strength of ML Me-PTCDI was obtained by analyzing the temperature-dependent differential reflectance on both quartz (Fig. 3c) and 275 nm SiO_2_/Si substrates (Fig. 3d) using transfer matrix method^29^ (see “Methods” section). We also obtained the absorption by measuring transmittance and reflectance of ML Me-PTCDI on quartz substrate at room temperature (supplementary Fig. 7). Strong light-matter interaction is evidenced by the large ΔR/R amplitude at resonance. Our modeling using two oscillators (corresponding to the superradient and single-molecule state, respectively) was in excellent agreement with experimental data. The derived peak absorption at the main resonance consistently exceeds 30% at 4 K (Fig. 3e, the variation is due to different as-grown sample quality), which is among the highest of any ML materials^30^. Importantly, the absorption is not constant but became progressively stronger at low temperature, which is a direct proof of enhanced oscillator strength *f* (Fig. 3e). Since we did not observe any sign of CT states from absorption^31^, it could only be explained by increasing exciton size in the J-aggregate. On the other hand, the single-molecule oscillator strength was much smaller than that of the main resonance (Supplementary Table 2). Further modeling on ΔR/R of multi-layer Me-PTCDI showed that the oscillator strength of the FE was about one order of magnitude smaller than ML (Supplementary Fig. 7, Supplementary Table 2). This is not surprising because the FE in multi-layers is heavily mixed with interlayer CT, leading to spatial separation of electrons and holes^28,32^.

We observed several other spectral changes to qualitatively support superradiance in ML. First, the exciton energy showed bathochromic shift on the order of *J* and saturated at low temperature (Fig. 3b–d). This behavior is consistent with the theory of 1D J-aggregate^33^, where energy shift is described by 2*J*·cos(π/(*N*_c_ + 1)) and approaches asymptotically to *2J* for large *N*_c_. We can rule out thermally induced strain or screening effects because the shift was not observed in multi-layers or monomers. Second, the PL exhibited strong increase of intensity and decrease of linewidth at low temperature (Fig. 3b), which was typical for superradiance^34^. The enhanced PL was also in line with the exciton oscillator strength from differential reflectance measurements. The brightening of PL was not observed in isolated monomers (Supplementary Fig. 8), indicating that coherent dipole interaction was responsible. We note that near-unity PLQY at room temperature is expected to maintain at low temperature, because the increased coherent length would lead to even shorter radiative lifetime. To validate this hypothesis, we plot integrated PL and absorption at 532/514 nm as a function of temperature (Supplementary Fig. 5c, d). As expected, they show the same scaling trend with temperature, indicating that the PLQY remains relatively constant in the whole temperature range.

## Theoretical modeling

**Fig. 4:**
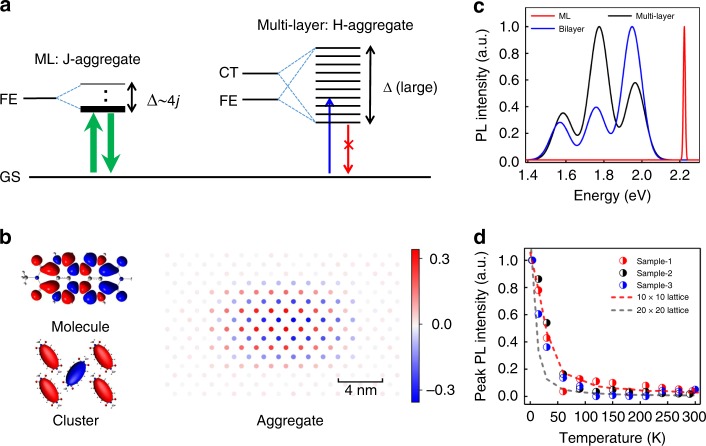
Theoretical modeling of layer-dependent excitonic properties. **a** Schematic illustration of the excitonic band diagram of ML and multi-layer Me-PTCDI. For ML, the lowest excited state is purely Frenkel and coherent. For bulk, the excitonic states are mixed Frenkel/CT and incoherent. **b** Frenkel transition density in Me-PTCDI molecule (upper left) and the spatial distribution of the lowest excited state in small cluster (lower left) and large aggregate (right) in ML. Red and blue in single molecule denote transition electrons and holes. The color scale in cluster/aggregate stands for the value of exciton wavefunction at each site. **c** Calculated PL spectra of ML, bi-layer and multi-layer Me-PTCDI crystals. **d** Simulated temperature dependence of the PL emission intensity for ML (using 10 × 10 and 20 × 20 lattice) with comparisons to three experimental data sets.

We rationalized our experimental results theoretically using a Hamiltonian including Frenkel, CT and their coupling with parameters derived from first-principle calculations^35,36^ (see Methods). As illustrated in Fig. 4a, the excitations in multi-layer perylene derivatives are mixtures of Frenkel and CT states due to the large interlayer electronic coupling (*D*_e_ for electrons, *D*_h_ for holes, Supplementary Table 1). The percentage of CT configurations is over 40% in bi-layer and quickly increased to the bulk value of ~75% at 6-layer (Supplementary Fig. 9c). On the other hand, the CT percentage in ML was negligible because the intralayer *D*_e_ and *D*_h_ were 1–2 orders of magnitude smaller, and the intralayer CT processes were 2 orders of magnitude slower (Supplementary Table 1, Supplementary Table 3). Indeed, we did not observe any evidence of CT states in ML absorption (Fig. 3a)^31^. As a result, the excitations in ML form a pure Frenkel J-band co mpletely separated from CT (Fig. 4a), which is a rare occasion in molecular systems^36^. Figure 4b plots the spatial distribution of the lowest excitation in ML Me-PTCDI, showing a delocalized envelop wavefunction. The coherent interaction of many Frenkel dipoles forms a giant superradiant transition dipole. The ellipsoidal shape of the wavefunction also explains the linear PL anisotropy in experiment (Fig. 3a).

Within above theoretical framework, the calculated PL spectra successfully reproduced all the main experimental features (Fig. 4c, Supplementary Fig. 9). The narrow PL emission and supressed vibronic progressions in ML were only possible to reproduce with extended coherence and without CT (Supplementary Fig. 9d–f). The coherent length could be estimated by fitting the temperature-dependent PL intensity, which is widely adopted in the community^37^. We find that a lattice size of 10–20 (*N*_c_ ~50–200) gives reasonably good agreement with experimental data (Fig. 4d, Supplementary Note 2), suggesting that *N*_c_ could reach the order of 100 at zero temperature. Compared to most biological aggregates and molecular crystals^13^, the coherence in ML Me-PTCDI is much extended both spatially and temporally (~30 ps versus sub-picosecond). This unique property is only accessible at 2D limit owing to reduced screening and suppression of CT.

## Light-emitting devices

**Fig. 5:**
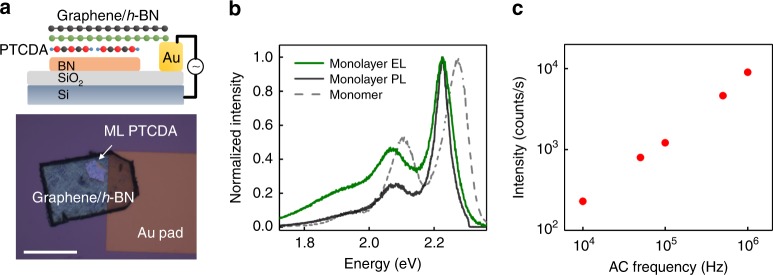
Transient light-emitting devices of ML perylene derivatives. **a** Schematic and optical image of the ML PTCDA transient light-emitting device for electrically exciting the coherent state. Scale bar: 50 μm. **b** t-EL and PL spectra measured on the same ML PTCDA device. The EL is excited using 20 V peak-to-peak square wave driving voltage. **c** Frequency dependence of EL intensity.

The fact that *J* is comparable to *k*_B_*T* at room temperature has important implications on device applications. To demonstrate this aspect, we fabricated electrically injected light-emitting devices on ML perylene derivatives (in this case PTCDA). To get carrier injection into ML PTCDA on insulating h-BN, a one-terminal, alternating current (AC) driven vertical device structure was employed (Fig. 5a)^38^. In this device, ML PTCDA/h-BN on 90 nm SiO_2_/Si p^++^ substrate was covered with few-layer CVD graphene as transparent source contact by micro-transferring, which was grounded through a metal bond pad (Fig. 5a). A ML CVD h-BN was inserted between graphene and PTCDA to prevent quenching of the EL. By applying an AC voltage to the Si backgate, alternating electrons and holes were injected into the ML both from the source contact and give transient electroluminescence (t-EL)^38^. Figure 5b shows the room-temperature t-EL spectrum under 20 V peak-to-peak square wave driving voltage. The peak at 2.224 eV can be clearly identified, which overlaps with the PL of ML. The EL peak shows ~50 meV red shift and reduced linewidth by a factor of 2 compared to single-molecule 0-0 Frenkel transition (at 2.274 eV). This suggests that the excitonic state can be excited electrically, which is an important step towards coherence-enabled device functions^13^. The EL intensity showed linear relationship with frequency as expected for AC driven devices (Fig. 5c)^38^. The linear trend persisted up to 20 MHz without intensity drop (limited by the loss in our measurement circuit), indicating that our EL devices could operate at much higher frequency. Considering the room-temperature PL lifetime ~27 ps (Fig. 2b), the intrinsic frequency limit should be above 30 GHz. Although the transient light-emitting devices are very preliminary, these results clearly demonstrate the potential of ML perylene derivatives for efficient and high-speed optoelectronic devices.

## Discussion

In conclusion, we report unusual optical properties emerging from a class of ML molecular J-aggregate of perylene derivatives. The photo-excitation is dominated by FE with coherently delocalized wavefunction, due to large unscreened dipole interactions and nearly complete elimination of CT processes. As a result, ML Me-PTCDI shows resonant absorption over 30%, bright PL emission (more than 2 orders of magnitude brighter than TMDs) and PLQY in the range of 60–100%. The temperature dependence of oscillator strength, exciton energy and linewidth provide evidence of superradiance. In addition, we demonstrate light-emitting devices on ML perylene derivatives and show that the superradiance is robust under electrical injection. These strongly emitting ML molecular crystals can be integrated with other 2D materials to form van der Waals heterostructures on-demand^39,40^, opening many new opportunities in high-performance optoelectronics.

## Methods

### vdW expitaxy growth of Me-PTCDI crystals on BN

We used mechanically exfoliated BN (hq graphene) on 275 nm SiO_2_/Si as the growth substrate without further cleaning. Before growth, the BN sheets were characterized by optical microscope and AFM to obtain the topological information. The growth of Me-PTCDI was carried out in a home-built tube furnace. The Me-PTCDI powder (Sigma Aldrich, 97% without further purification) and substrates were placed at the center and downstream in the furnace, respectively. After evacuating the chamber to ~3 × 10^−3^ Pa, the furnace was heated to 210–260 °C to start growing Me-PTCDI crystals. The number of layers can be controlled by the heating temperature, growth duration and substrate position.

### AFM and Raman spectroscopy

AFM (both regular and high-resolution) was performed by Asylum Cypher under ambient condition. We used Asylum TR400PB tips for high-resolution AFM. Raman spectroscopy was performed by Princeton Instruments SP-2560 spectrometer coupled to a silicon CCD camera (Princeton Instruments PYL-1300BXD). We used a 488 nm laser as excitation.

### TEM measurement

The selected-area electron diffraction (SAED) pattern was collected on a 200 kV FEI Tecnai F20 transmission electron microscope (TEM). The TEM samples were prepared by transferring few-layer BN sheets exfoliated on SiO_2_/Si substrate onto Cu TEM grid with carbon film, using the process described in reference^41^ and growing organic films on transferred BN. As this organic crystal sample is highly electron beam sensitive, the SAED pattern was acquired with a short exposure time of 0.01 s to prevent from the beam damage. The SAED simulations were calculated using the multislice method with the Kirkland code^42^.

### Optical spectroscopies

The PL images were collected using an optical microscope (Olympus BX51M). The samples were illuminated by a 450 nm LED and the PL images were collected by a colored CCD camera. The excitation light was filtered by a long-pass filter in front of the CCD.

Temperature-dependent PL measurements were done inside an optical cryostat (Montana Cryostation S50) coupled to Horiba LabRAM HR800 system. We used 532 nm laser as excitation. The base temperature of the cryostat was 4 K. The PL were collected by a 50 × objective (NA = 0.45). Polarization-dependent PL was tested by rotating a linear polarizer between the sample and detector, while keeping the incident laser polarization.

For TRPL measurement, the excitation light is generated by a mode-locked Ti:sapphire laser with an optical parametric oscillator. The laser pulse width is on the order of 200 fs. The light is focused onto the sample by a Zeiss 50× objective. The emission signal, detected in the reflection configuration, is passed through a bandpass filter with a bandwidth of 20 nm and collected by a synchroscan Hamamatsu streak camera (C10910-02), whose overall time resolution is 2 ps.

For absorption measurements, the sample was placed in cryostat chamber (OXFORD INSTRUMENTS_MicrostatHires) cooled by liquid helium. The broadband radiation from a halogen lamp covering the spectral range from 450 to 900 nm was radiated on the sample without focus. The transmission signal was collected by 50× objective (NA = 0.7) and analyzed by a liquid nitrogen cooled silicon CCD detector with a spectrograph. Absorption signal was determined by measured normalizing the transmission signal from the sample on BN/fused silica to that from the bare fused silica.

### PLQY measurement

We use a dedicated setup at UC Berkeley to measure PLQY. The same instruments and procedures were used as in several previously papers^22,43^. Below we describe the details of the measurement (Supplementary Fig. 5a). As excitation source, an Ar + laser (Lexel 95) with 514 nm line was utilized in steady-state PL and the power density was adjusted by neutral density filters and simultaneously monitored by photodiode power sensor (ThorLabs S120C). The ratio of laser power on the diode to incident power onto sample was around 50 so that the low laser power can be accurately measured. A Si CCD detector (Andor iDus BEX2-DD) on an *f* = 340 mm spectrometer with a 150 g mm^−1^ grating was used to collect the steady-state PL spectra and the dark background of CCD was measured and subtracted each time from the acquired signal (Supplementary Fig. 5a, left). A 50 × MD Plan (Olympus) objective lens (numerical aperture, 0.55) was used for all measurements. For PLQY and room temperature quantitative absorption measurements, we used quartz substrates to avoid optical interference from Si/SiO_2_ substrates. Calibration for the external sample PL efficiency was performed using the wavelength-dependent instrument function which characterized the collection efficiency of the instrument. This calibration process was previously described in our work and have below 15% error when cross calibrated with a Si photodiode or using a dye solution of unity quantum yield^22^. In brief, the instrument function is measured using a near ideal diffuse reflector as the source of calibration. The internal PLQY was extracted from the measured external quantum efficiency using the quantitative absorption at the pump wavelength and by the fraction of light within escape cone (1/4*n*^2^, where *n* is the refractive index of the medium).

To quantitatively calculate PLQY, we need to measure the absolute absorption of ML Me-PTCDI at 514 nm. Here the absorption is measured by 100%-T-R, where the transmittance and reflectance are individually determined using lock-in detection (Supplementary Fig. 5a, right). The laser was focused on the sample using a 50× objective, the reflected light was collected via the same objective and the transmitted light was collected by a 20× objective. The system was calibrated using quartz and silver as reference transmission and reflectance standards. The reported generation rates (steady-state measurements) and initial carrier densities (time-resolved measurements) are calculated from the number of incident photons per unit area and the absorption.

### ACEL device

The ACEL device of PTCDA is fabricated by multiple transfer steps, where one layer of graphene and one layer of h-BN is put on top of the ML molecular crystal. Specifically, the device was fabricated by two methods and gave similar result, indicating the molecular monolayer can survive the transfer process.

Method 1: An array of Au pads (75 μm × 75 μm square) is fabricated on 90 nm SiO_2_/Si p++ substrates by photolithography. Using PMMA pad (~400 × 400 μm) to sequentially pick up CVD multi-layer graphene (on Si/SiO_2_ substrate), CVD monolayer h-BN (on Si/SiO_2_ substrate), and the ML on BN, and put down the stack on pre-patterned substrate (metal electrode on Si/SiO_2_) so that the top graphene layer overlap with both the metal pad electrode and the ML. In a typical pick up process, the PMMA is first put on top of the target layer (graphene, BN, or ML on BN) by a micro-tip, and the substrate is heated at 180 °C for 90 s so that the PMMA would pass the glass transition temperature and bound to the target layer. Then an engraver and a micro-tip is used to decouple the PMMA from the substrate with the target layer and transfer the PMMA stack to another target layer. In this process the ML layer survived 180 °C heating during the dry transfer.

Method 2: To reduce sequential dry transfers and increase device fabrication yield, the same dry transfer processed was used to first pick up a monolayer CVD h-BN, put down on ML-BN, then pick up this stack and transfer to the patterned substrate. Then dichloromethane is used to dissolve the PMMA of this stack, and another PMMA is used to pick up the graphene contact and put down on this substrate so that the graphene covers the electrode and the CVD BN-ML-BN stack. The device fabricated in this method behave similarly to method 1, and in this process the heterostructure survives the dichloromethane solvent treatment.

In this device, the graphene on top bridge the Au pad and the PTCDA and allows carrier injection into the PTCDA ML. The CVD monolayer h-BN and CVD multilayer graphene was purchased from Graphene Supermarket.

### Theoretical model and derivation of the Hamiltonian parameter**s**

To qualitatively describe the nature of the excitonic states in Me-PTCDI, we employ the widely used electronic-vibrational model for molecular excitonic systems^35^, in which the electronic part is

1 $$\begin{array}{l}H_{\mathrm{el}} = \mathop {\sum}\limits_m {\omega _{{\mathrm{FE}}}} \left| m \right\rangle \left\langle m \right| + \mathop {\sum}\limits_{\left\langle {mn} \right\rangle } J \left| m \right\rangle \left\langle n \right| + \mathop {\sum}\limits_{n_ + ,n_ - } {\omega _{{\mathrm{CT}}}} |n_ + ;n_ - \rangle \langle n_ + ;n_ - |\\ {\hskip 34pt}+ \mathop {\sum}\limits_{n_ + ,\langle n_ - ,n_ - ^\prime \rangle } {t_{\mathrm{e}}} |n_ + ;n_ - \rangle \langle n_ + ;n_ - ^\prime | + \mathop {\sum}\limits_{\langle n_ + ,n_ + ^\prime ,n_ - \rangle } {t_{\mathrm{h}}} |n_ + ;n_ - \rangle \langle n_ + ^\prime ;n_ - |\\ {\hskip 14pt}+ \mathop {\sum}\limits_{\langle mn\rangle } {D_{\mathrm{e}}} \left( {\left| n \right\rangle \left\langle {n;m} \right| + h.c.} \right) + \mathop {\sum}\limits_{\langle mn\rangle } {D_{\mathrm{h}}} \left( {\left| n \right\rangle \left\langle {m;m} \right| + h.c.} \right),\end{array}$$

where the first two terms represent Frenkel states, the next three terms represent CT states, and the last two terms are the coupling between them. The excited eigenstates are linear combinations of the FE and CT configurations, and the coefficients depends on their energy difference $({\mathrm{\Delta }}_{{\mathrm{FE}} - {\mathrm{CT}}} = \left| {\omega _{{\mathrm{FE}}} - \omega _{{\mathrm{CT}}}} \right|)$ as well as the off-diagonal coupling terms (*J* for interaction between FE ones, *t*_e_ and *t*_h_ for charge hopping integral among CT ones, and *D*_e_ and *D*_h_ for coupling between FE and CT states).

The vibrational part is

2 $$	\begin{array}{l}H_{{\mathrm{vib}}} = {\upomega}_{{\mathrm{vib}}}\mathop {\sum}\limits_n {b_n^\dagger } b_n + {\upomega}_{{\mathrm{vib}}}\mathop {\sum}\limits_n {\{ \lambda (b_n^\dagger + b_n) + \lambda ^2\} } |n\rangle \langle n|\\ \ \ 	{\hskip -202pt}+ {\upomega}_{{\mathrm{vib}}}\mathop {\sum}\limits_{n_ + ,n_ - } {\{ \lambda _ + (b_{n_ + }^\dagger + b_{n_ + }) + \lambda _ - (b_{n_ - }^\dagger + b_{n_ - }) + \lambda _ + ^2 + \lambda _ - ^2\} } |n_ + ;n_ - \rangle \langle n_ + ;n_ - |\end{array}$$

The first term is the pure vibrational energy. And $b_n^\dagger$ creates a vibrational excitation with energy *ω*_vib_ on site *n*, whereas *b*_*n*_ annihilates the same. The last two terms are the electron-phonon coupling, and *λ*^2^, $\lambda _ + ^2$ and $\lambda _ - ^2$ represent Huang-Rhys factors^44^ for exciton, cation and anion respectively.

The Hamiltonian parameters shown in Supplementary Table 1 are derived from DFT and time-dependent density functional theory (TDDFT) calculations of molecular monomers or dimers at M06-2×/6-31G level by using GAUSSIAN 09 program^45^.

*ω*_0−0_ and *ω*_vib_ are extracted from the vibronic spectra calculations by using the Franck-Condon method after a TDDFT calculation for the pure electronic excitation for the molecular monomer. *λ*^2^, $\lambda _ + ^2$ and $\lambda _ - ^2$ are calculated by using Duschinsky rotation method with DUSHIN program^46^. The excitonic couplings *J* are calculated using the Generalized Mulliken-Hush (GMH) method^47^. The charge-transfer integrals (*D*_e_ and *D*_h_) are computed as the Fock matrix elements between frontier molecular orbitals of the neighboring molecules.

For computing the calculated time scale for inter-molecular electron/hole charge transfer in Me-PTCDI crystals, we use the Marcus theory to calculate the intermolecular CT rate (*k*)

3 $$\frac{1}{t} = k = \frac{{2{\uppi}}}{\hbar }\frac{{\left| {H_{{\mathrm{ab}}}} \right|^2}}{{\sqrt {4{\uppi}\lambda k_{\mathrm{B}}T} }}{\mathrm{exp}}\left[ { - \frac{{\left( {\lambda + {\mathrm{\Delta }}G} \right)^2}}{{4\lambda k_{\mathrm{B}}T}}} \right].$$

Here *T* is the temperature, *k*_B_ is the Boltzmann constant, *H*_ab_ is the electronic coupling (*D*_e_ and *D*_h_ for electron and hole respectively), *λ* is the reorganization energy and Δ*G* is free energy (in this case, we use Δ*G* = 0). We calculated the reorganization energy values for the electron (*λ*_−_) and hole (*λ*_+_) carriers using the following equation:

4 $$\lambda _{ - / + } = \left( {E_{ - / + }^ \ast - E_{ - / + }} \right) + \left( {E_{ - / + }^{ \ast \ast } - E_0} \right)$$

where $E_{ - / + }^ \ast$ is the vertical energy of negatively/positively charged molecule at the geometry of the optimized neutral molecules, $E_{ - / + }$ is the optimized energy of negatively/positively charged molecules, $E_{ - / + }^{ \ast \ast }$ is the neutral energy of molecules at the geometry of optimized negatively/positively charged species, and *E*_0_ is the ground-state energy at the optimized geometry of the neutral molecule.

### Transfer matrix method of calculating absorption

We model the dielectric function of Me-PTCDI by the Lorentz model. The dielectric function is given by

5 $${\it{\epsilon }}\left( E \right) = {\it{\epsilon }}_\infty + \mathop {\sum}\limits_{i = 1}^N {\frac{{f_iE_i^2}}{{E_i^2 - E^2 + i\gamma _iE}}}$$

where *E*_*i*_, *γ*_*i*_ and *f*_*i*_ are the resonance energy, damping rate and oscillator strength of the *i*^*th*^ exciton, respectively. ${\it{\epsilon }}_\infty$ is the background dielectric constant that accounts for the contributions from resonances at higher energies.

Given the dielectric function of Me-PTCDI, the differential reflectance spectra then can be directly calculated using the transfer matrix method. Here we use the BN/quartz substrate as an example. In general, the transfer matrix at a dielectric boundary between two media of refractive indices *n*_1_ and *n*_2_ are given by

6 $$M_{12} = \frac{1}{{n_1 + n_2}}\left( {\begin{array}{*{20}{c}} {n_2 + n_1} & {n_2 - n_1} \\ {n_2 - n_1} & {n_2 + n_1} \end{array}} \right)$$

And the propagation matrix within a uniform layer of refractive index *n*_*l*_ and thickness *d*_*l*_ is given by

7 $$P_l = \left( {\begin{array}{*{20}{c}} {e^{in_lkd_l}} & 0 \\ 0 & {e^{ - in_lkd_l}} \end{array}} \right)$$

where *k* is the wave vector in free space.

The transfer matrices for the Me-PTCDI layer on the substrate, and the pure substrate then can be written as

8 $$M_{\mathrm{P}} = M_{{\mathrm{air}},{\mathrm{p}}}P_{{\mathrm{PTCDI}}}M_{{\mathrm{p}},{\mathrm{BN}}}P_{{\mathrm{BN}}}M_{{\mathrm{BN}},{\mathrm{q}}} = \left( {\begin{array}{*{20}{c}} {1/t_{\mathrm{p}}} & {r_{\mathrm{p}}^ \ast /t_{\mathrm{p}}^ \ast } \\ {r_{\mathrm{p}}/t_{\mathrm{p}}} & {1/t_p^ \ast } \end{array}} \right)$$

9 $$M_{{\mathrm{sub}}} = M_{{\mathrm{air}},{\mathrm{BN}}}P_{{\mathrm{BN}}}M_{{\mathrm{BN}},{\mathrm{q}}} = \left( {\begin{array}{*{20}{c}} {1/t_{{\mathrm{sub}}}} & {r_{{\mathrm{sub}}}^ \ast /t_{{\mathrm{sub}}}^ \ast } \\ {r_{{\mathrm{sub}}}/t_{{\mathrm{sub}}}} & {1/t_{{\mathrm{sub}}}^ \ast } \end{array}} \right)$$

The differential spectrum Δ*R*/*R* then is given by

10 $$\frac{{\Delta R}}{R} = \frac{{\left| {r_{\mathrm{p}}} \right|^2 - \left| {r_{{\mathrm{sub}}}} \right|^2}}{{\left| {r_{{\mathrm{sub}}}} \right|^2}}$$

The dielectric function of Me-PTCDI then can be obtained by fitting the differential reflectance spectrum.

For all the samples, the thickness of the h-BN was measured by AFM and accounted for in the modeling. In addition, the reference point was measured on the same BN. This was achieved by covering part of the BN by Au pad and peeling it off after Me-PTCDI.

## Supplementary information

Supplementary Information

## Data Availability

The data that support the findings of this study are available from the corresponding author upon reasonable request.
