# Supplementary material for: Strong optical response and light emission from a monolayer molecular crystal
Source: Nat Commun. 2019 Dec 6;10:5589. doi: 10.1038/s41467-019-13581-9 (PMC6897925; doi:10.1038/s41467-019-13581-9)
Supplement: Supplementary file 1 — Supplementary Information [file 41467_2019_13581_MOESM1_ESM.pdf]

Supplementary Information

Strong optical response and light emission from a monolayer molecular crystal

Zhao *et al*.

Supplementary Note 1: Experimental results on PTCDA and PTCDI

In addition to Me-PTCDI, we investigated the optical properties of two sister molecules, 3,4,9,10-perylene-tetracarboxylic dianhydride (PTCDA) and 3,4,9,10-perylene-tetracarboxylic diimide (PTCDI) (Supplementary Fig. 10a). Similar to Me-PTCDI, in ML PTCDA and PTCDI we observed the sharp PL peak near 2.2 eV (Supplementary Fig. 10b). The PL peak of ML PTCDA was red-shifted from monomer 0-0 Frenkel transition (Supplementary Fig. 10c). For multi-layer PTCDA, the PL peak is centered near 1.8eV. This leads to the distinct green and red color under PL image for ML and multi-layer PTCDA, respectively (Supplementary Fig. 1).

We also measured temperature-dependent PL and absorption of ML PTCDA (Supplementary Fig. 10d,e) and reproduced all the major findings in ML Me-PTCDI: bathochromic shift, enhanced intensity and reduced linewidth of PL at low temperature; absorption from the delocalized FE emerging at low temperature. The delocalized FE absorption in PTCDA is not as strong as Me-PTCDI, which precludes us to perform quantitative analysis. This indicates that the exciton wavefunction size in PTCDA is not as large as Me-PTCDI, probably due to the reduction of excitonic coupling due to the more perpendicular arrangement of two molecules in the unit cell.

Supplementary Note 2: Calculation of coherent size in monolayer

In order to estimate the chromophores involved in ML super-radiance, we calculate PL spectra in ML for different cluster sizes and compare to the experimental data at room temperature (Supplementary Fig. 9b). By taking the experimental monomer PL spectrum as a reference and varying the 2D cluster size, we find that a 3×3 lattice gives best agreement with experimental ML PL spectrum at room temperature, with three main characters being compared, i.e. energy shift from monomer to monolayer, full width at half maxima (FWHM) and ratio of intensities for peak 0-1 and peak 0-0. So the coherent size at room temperature can be estimated to be about 9. By considering the super-radiant rule for PL intensity ($I \propto N_{\mathrm{c}}$), the coherent size of excitons in ML at 4K is estimated to be about 225 since $\frac{I_{3\mathrm{K}}}{I_{300\mathrm{K}}}$ is experimentally determined to be around 25.

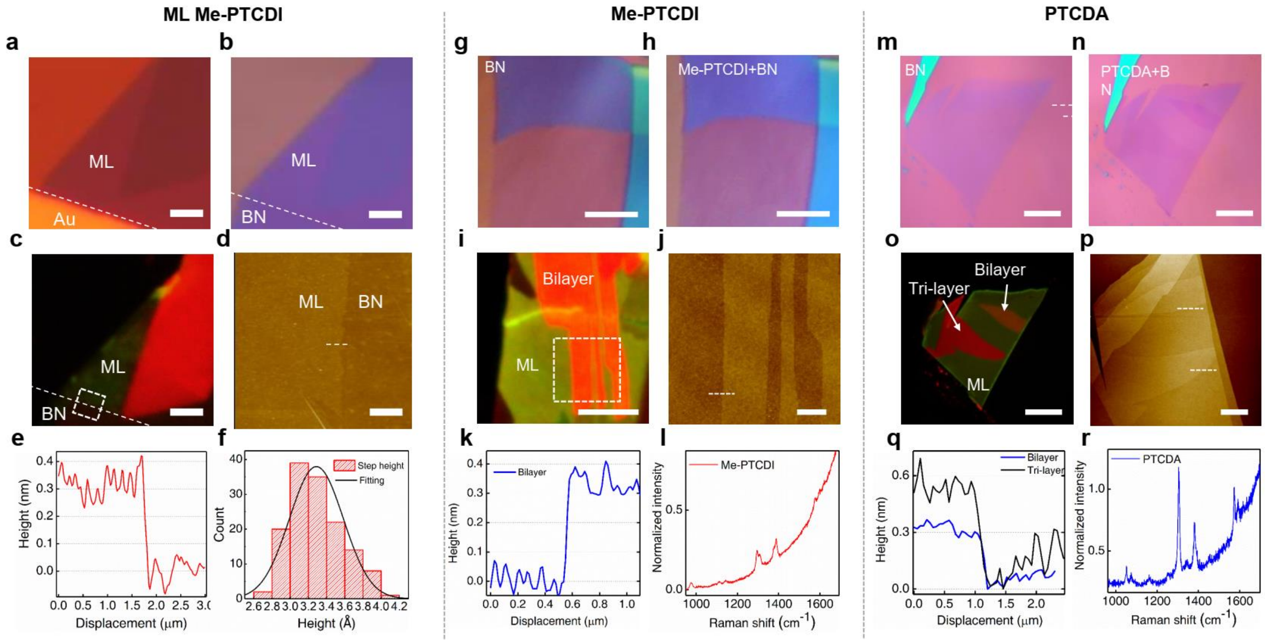

Supplementary Fig. 1 | Characterizations more Me-PTCDI and PTCDA samples. a, Optical microscope images of a half ML Me-PTCDI and half Au film sample. **b**, Optical microscope images of a half ML Me-PTCDI and half BN sample after peeling up Au film. **c**, The PL image of the same sample, where the green regions are ML and dark regions are BN/SiO_2. Scale bars: 5 μm. **d**, AFM images of the same area marked by the upper dashed box in (**c**) Scale bars: 1 μm. **e**, The height of the step between ML/BN and BN in (**c**). **f**, Statistical analysis of step height in multi-layer samples. The distribution curve shows the height of each layer at ~0.33 nm. Optical microscope images of a BN **g**, before and **h**, after Me-PTCDI growth. **i**, The PL image of the same sample, where the green regions are ML and red regions are multilayer. Scale bars: 10 μm. **j**, AFM images of the same area marked by the upper dashed box in (**i**) after Me-PTCDI growth. Next to the ML is a bilayer Me-PTCDI, according to the height difference of the step. Scale bars: 2 μm. **k**, The height of the step between ML and bilayer in (**j**). **l**, Raman spectrum of the Me-PTCDI region in (**i**) excited by 488 nm laser. The increasing background at large Raman shift is due to the PL of ML. Optical microscope images of a BN **m**, before and **n**, after PTCDA growth. Scale bars: 10 μm. **o**, PL image of the same sample, where the green regions are ML and red regions are bilayer and tri-layer. **p**, AFM images of the sample after PTCDA growth. The bilayer and trilayer regions are marked according to the height difference from the ML region. Scale bars: 5 μm. **q**, The height of the step between ML and bilayer in (**p**). **r**, Raman spectrum of the ML PTCDA excited by 488 nm laser.

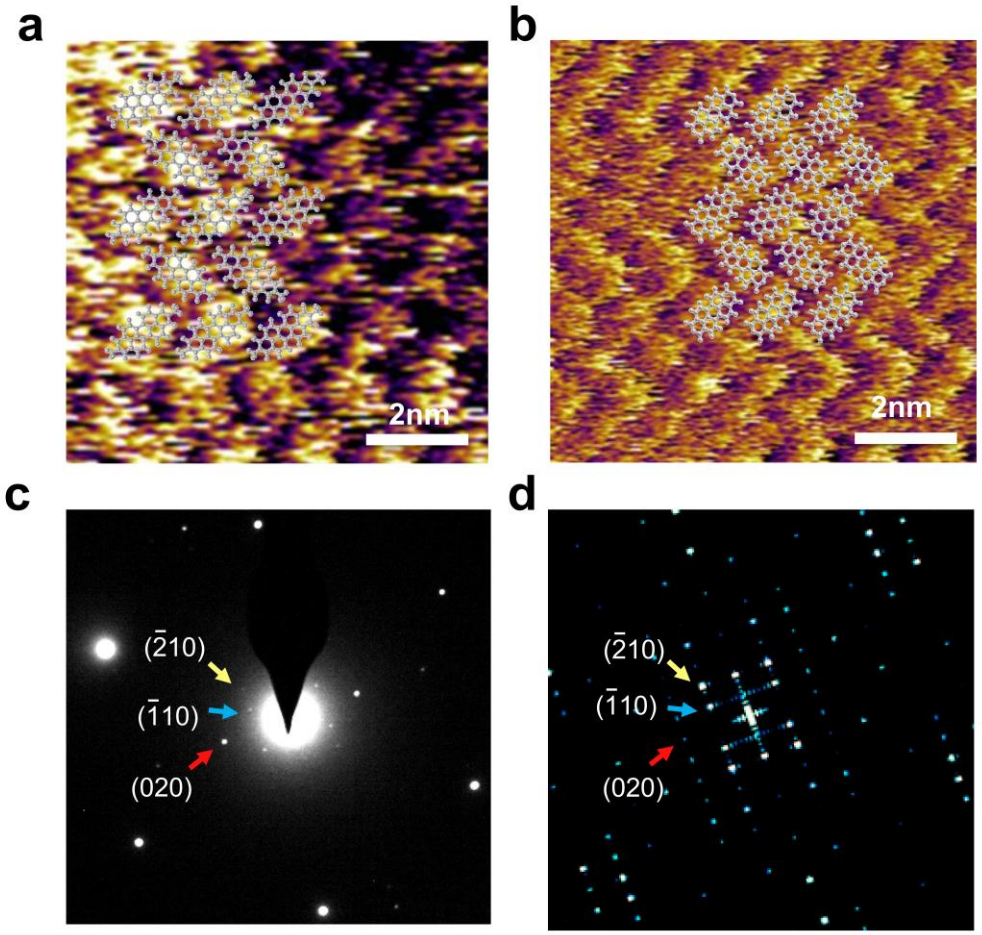

Supplementary Fig. 2 | High-resolution AFM and SAED characterizations. a and **b**, High-resolution AFM images of multi-layer Me-PTCDI (**a**) and PTCDA (**b**), both consistent with the herringbone-type packing. However, due to the thermal drift at ambient conditions, high-resolution AFM cannot provide accurate measurement of lattice constants. **c**, SAED pattern of Me-PTCDI films grown on h-BN. The diffraction spots of h-BN were clearly observed and used as references. Close to the center a set of rectangular diffraction spots corresponding to Me-PTCDI was observed. The plane indices were marked on several spots. According to this experimental diffraction pattern, the lattice structure shown in Fig. 1a was optimized by DFT calculations. **d**, Simulated diffraction pattern using the lattice structure of Fig. 1a, in good agreement with the experimental SAED pattern.

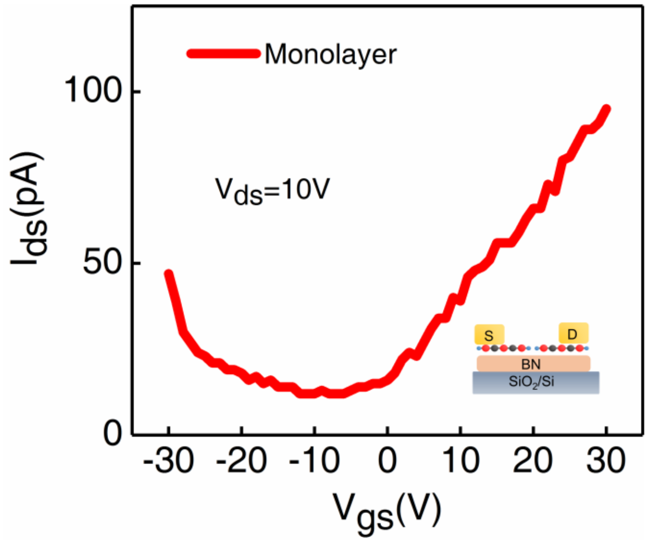

Supplementary Fig. 3 | Transfer characteristics of monolayer Me-PTCDI FET.

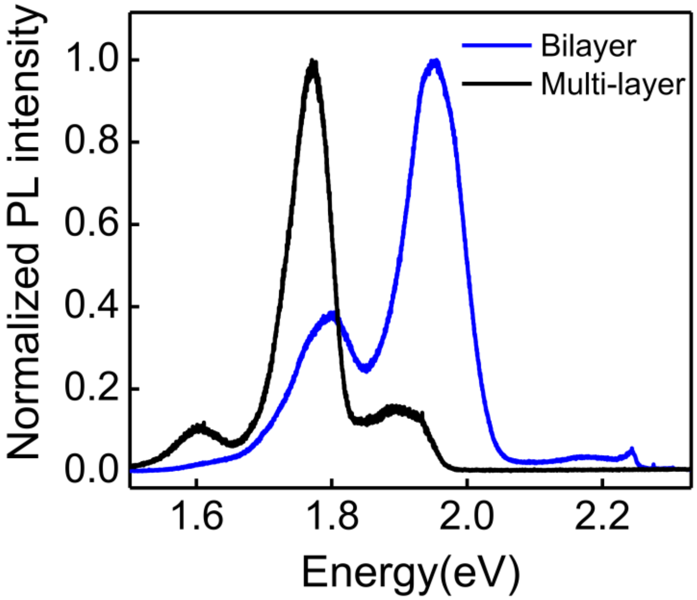

Supplementary Fig. 4 | PL of bi-layer and multi-layer Me-PTCDI. The PL of bi-layer also has three broad peaks close to that of multi-layer, which leads to similar red luminescence in PL image (see Supplementary Fig. 1). The resemblance of bi-layer and multi-layer PL spectra suggests that the optical excitation is still dominated by inter-layer CT at bi-layer. The different intensity ratio among the three peaks is reproduced by our DFT calculations and is due to the different percentage of CT state.

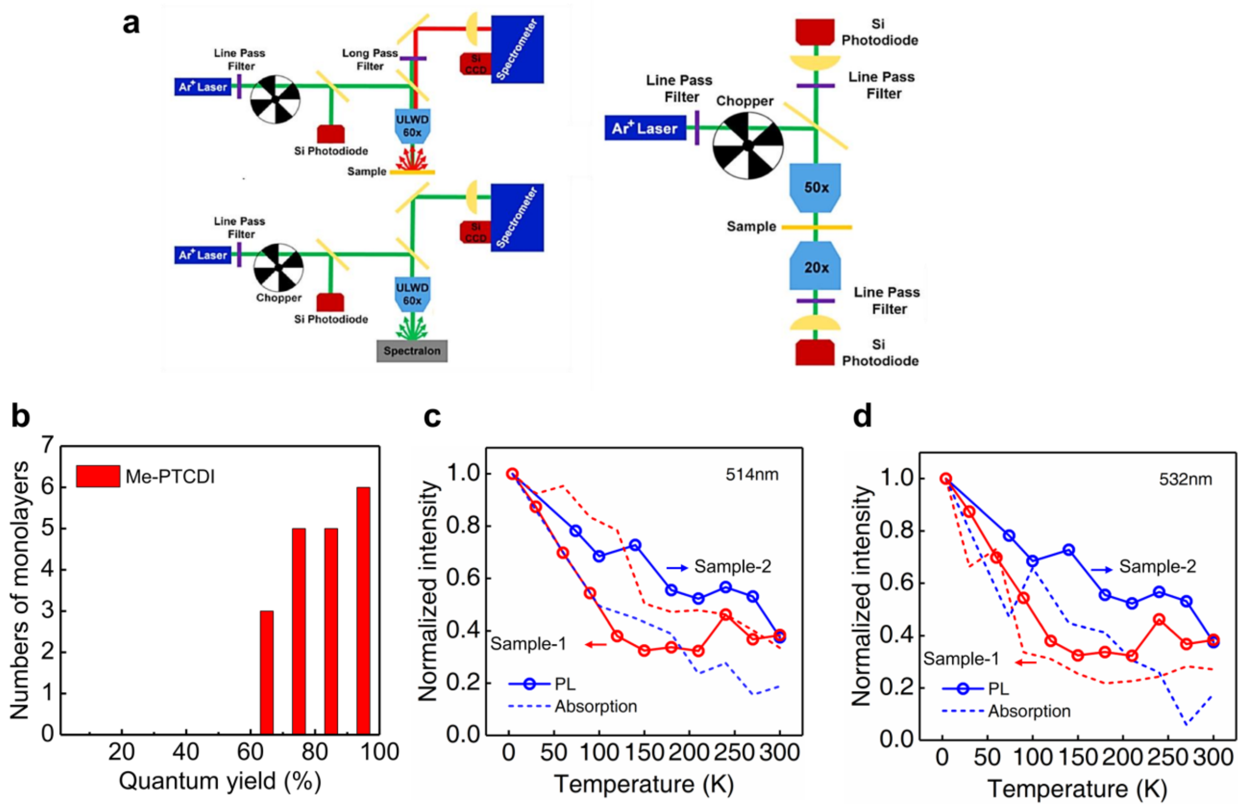

Supplementary Fig. 5 | PLQY measurement. a, Schematic of setup for PLQY measurement. (Left) Optical setup used to measure PL spectrum. (Right) Optical setup used for calibrating the absolute collection efficiency via a near ideal diffuse reflector. **b,** Histogram of PLQY of 19 monolayer Me-PTCDI samples measured moderate pump power (1.5 W cm^{-2}). The PLQY shows some variations due to the different sample quality as grown. However, PLQY>90% has been reproducibly observed in about 1/3 of the samples. **c** and **d,** Normalized PL and absorption (at 514nm (**c**) and 532nm (**d**)) of two ML Me-PTCDI samples (red and blue) as a function of temperature. The absorption was derived by transfer matrix modeling of ΔR/R measurements. The absorption at the two wavelengths was selected because the PL was excited at 532nm and the PLQY was measured at 514nm. The PL and absorption show the same scaling trend with temperature, indicating that the PLQY was maintained at low temperature.

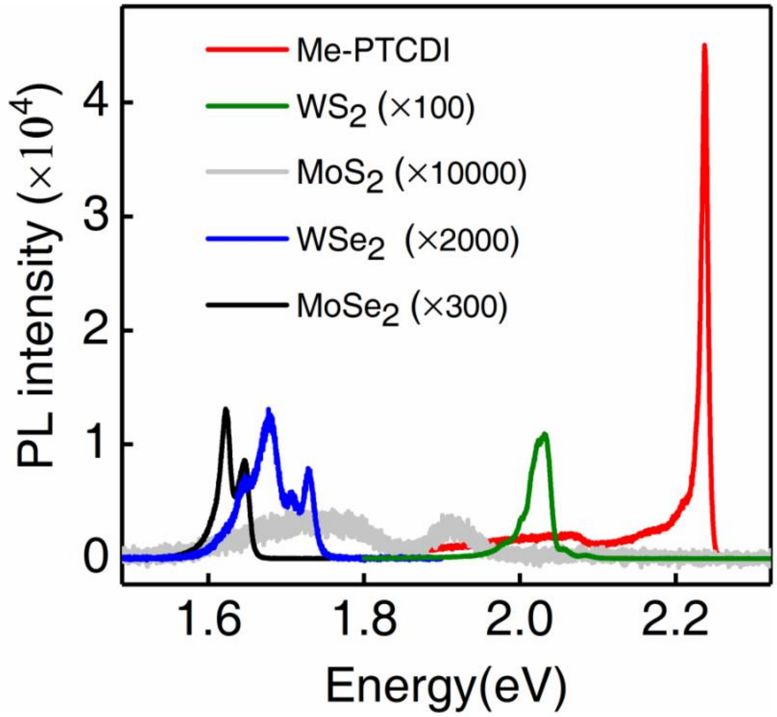

Supplementary Fig. 6 | Quantitative comparison of PL between ML Me-PTCDI and ML TMDs. The PL spectra were taken at 4K under 50 μW 532nm laser excitation and 50 ms integration time. The PL spectra of TMDs are multiplied for clarity.

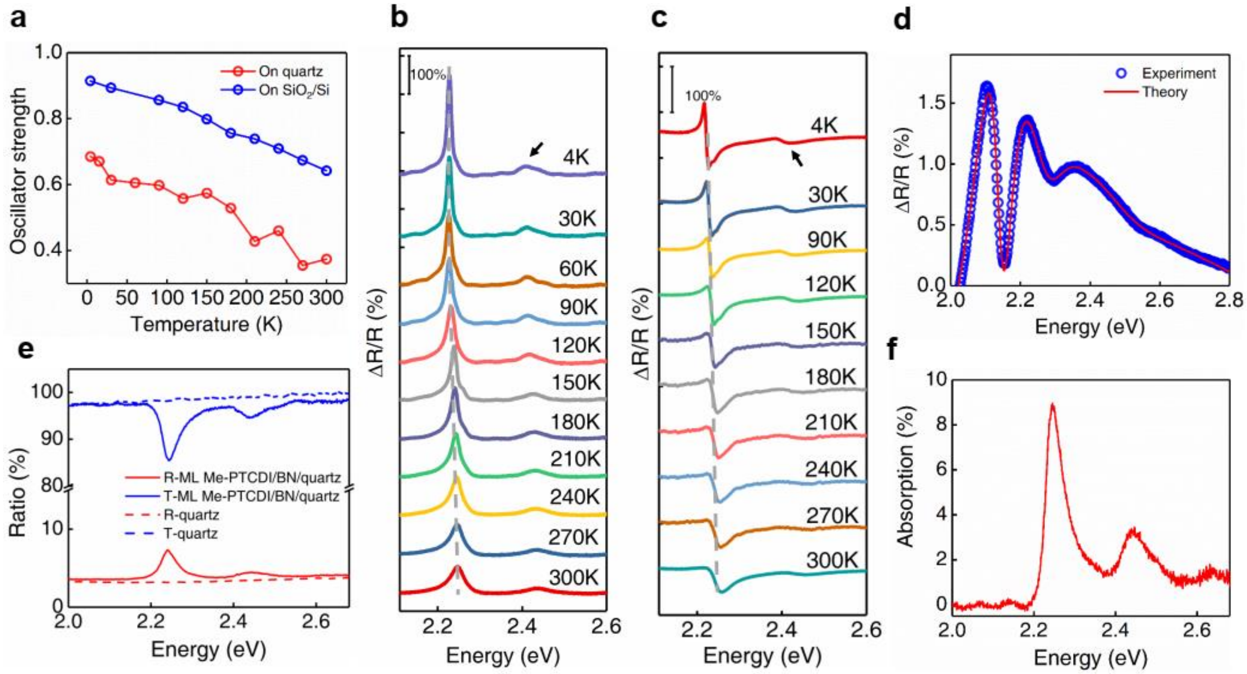

Supplementary Fig. 7 | Oscillator strength and differential reflectance of Me-PTCDI. a, Temperature dependent oscillator strength of ML Me-PTCDI on quartz (red) and SiO_2/Si (blue). **b** and **c,** Temperature-dependent absorption of the same sample in (**a**), (on quartz (**b**) and on SiO_2/Si (**c**)). **d,** The ΔR/R spectrum of multilayer Me-PTCDI on SiO_2/Si substrate. The resonance energies were in good agreement the literature[1]. According to the reference 1, the lowest absorption peak near 2.1 eV is mostly of CT characteristics and the second peak near 2.25 eV is the lowest Frenkel state. Using the transfer matrix method[2,3] , we model the ΔR/R using four oscillators and obtain the oscillator strength of the Frenkel state in multilayer to be 0.072, which is one order of magnitude less than monolayer. **e**, Transmittance and reflectance from quartz (dashed lines) and ML Me-PTCDI/BN on quartz (solid lines), respectively. **f**, Absorption of ML Me-PTCDI by 1-T-R.

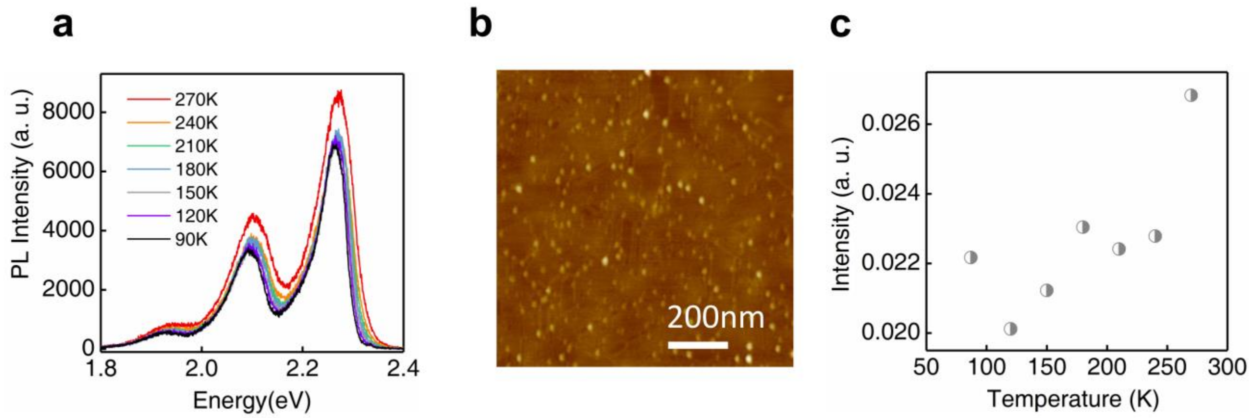

Supplementary Fig. 8 | PL of Me-PTCDI diluted monomers spin cast on BN. a, PL spectra of diluted monomers at different temperatures showing little change of intensity. **b**, AFM image showing isolated Me-PTCDI molecules on BN substrate. Scale bar: 200 nm. **c**, Integrated PL intensity as a function of temperature obtained from (**a**).

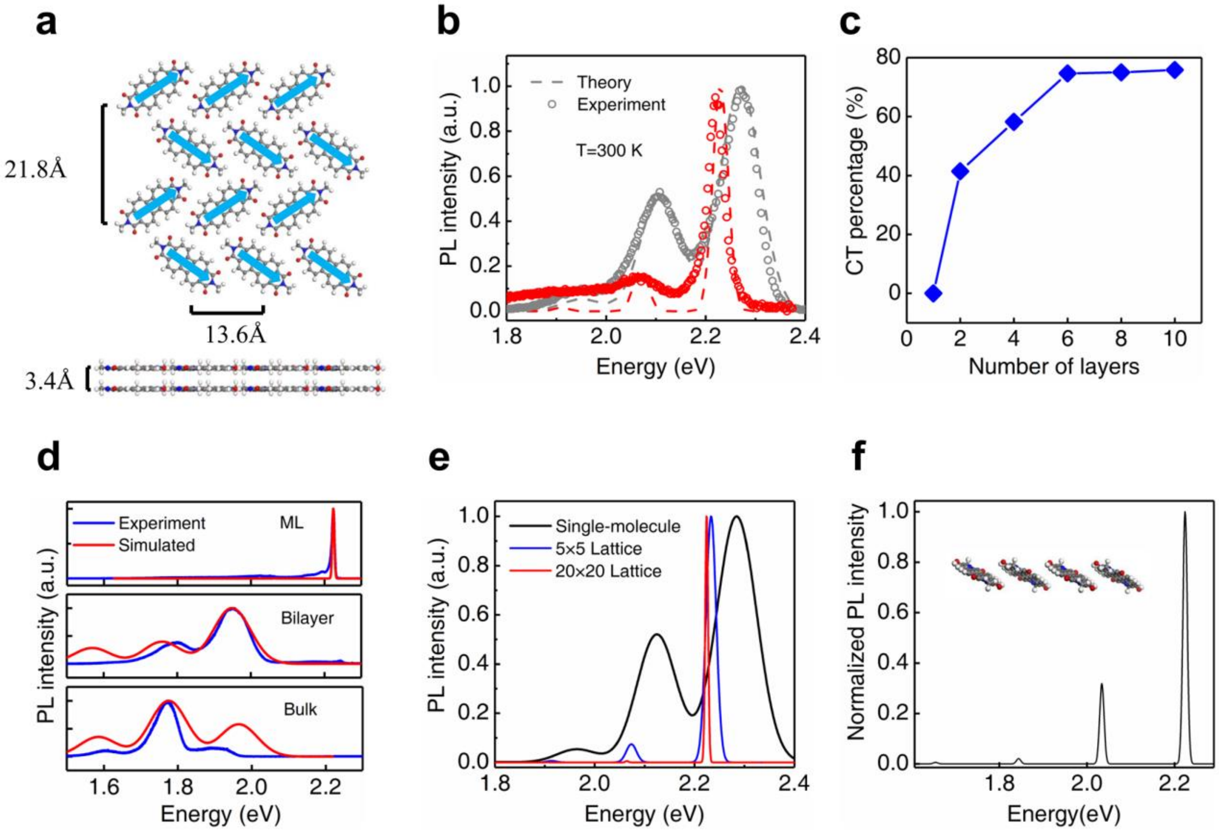

Supplementary Fig. 9 | Theoretical calculation of layer-dependent optical properties of ML Me-PTCDI. **a**, Structures of ML Me-PTCDI. **b**, Experimental PL spectra of Me-PTCDI ML (red symbols) and monomer (grey symbols) along with theoretical calculations (dashed lines). For ML, the calculation uses 3×3 lattice with N_c~10. The FWHM of 0-0 peak is 84.7 (monomer, experiment), 94.1 (monomer, theory), 37.4 (ML, experiment) and 35.3 (ML, theory) meV. Ratios of vibronic peaks (0-1/0-0) is 0.519 (monomer, experiment), 0.520 (monomer, theory), 0.151 (ML, experiment) and 0.181 (ML, theory). The experimental red-shift of peak energy in ML is also captured by theoretical calculation. **c**, The calculated CT percentage in the lowest excitation as a function of thickness. **d**, Simulated PL spectra compared with experiment. **e**, Calculated PL spectra of ML Me-PTCDI using different sized clusters. **f**, Calculated PL spectrum of ML (100) plane[4] of Me-PTCDI, which disagrees with experiments. Such a disagreement is mainly caused by the significant intralayer CT contributions within (100) plane, which is evident from the molecular packing as shown in the inset.

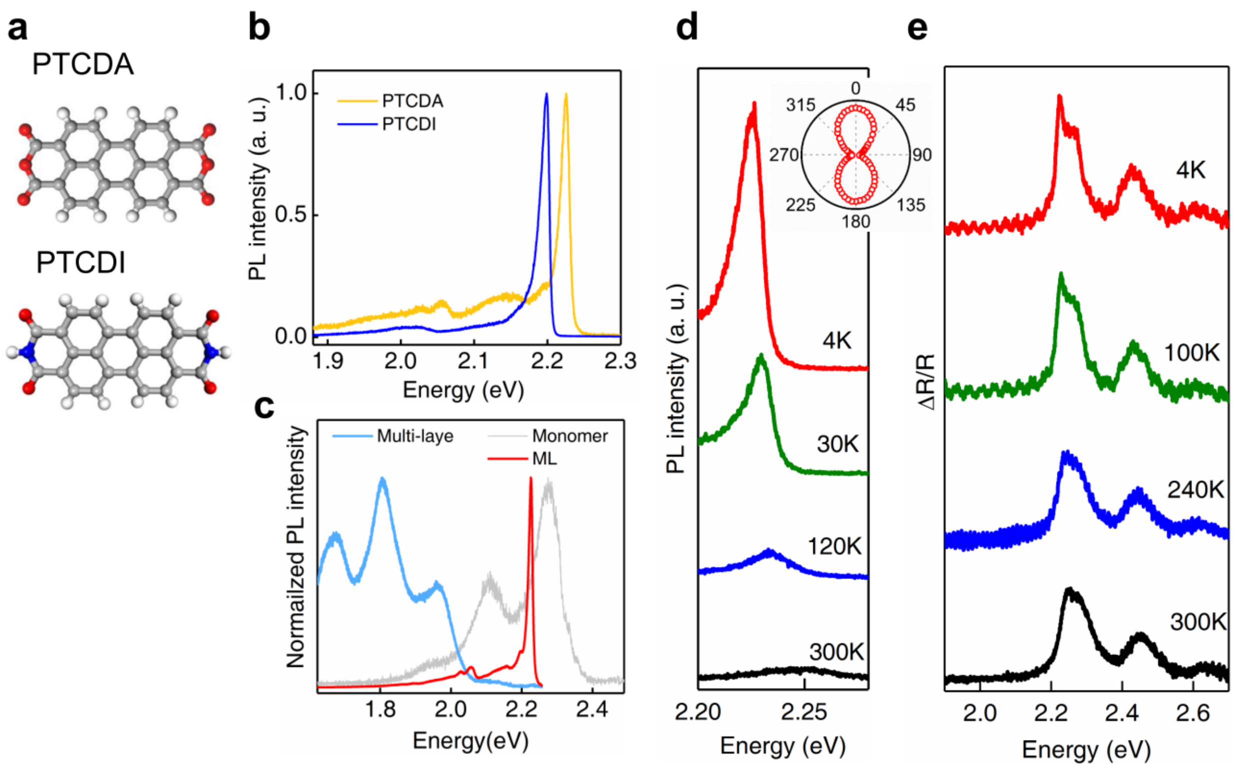

Supplementary Fig. 10 | Optical properties of other perylene derivatives. a, Molecular structures of PTCDA and PTCDI. **b**, Normalized PL spectra of ML PTCDA and PTCDI at 4 K. The two materials show similar spectrum with slightly different peak energy. **c**, Normalized PL spectra of monomer, ML, monomer and multi-layer PTCDA samples at 4 K, excited by 532 nm laser. **d**, Temperature-dependent PL spectra of ML PTCDA. Inset: polarization-dependent PL intensity showing 180° period. **e**, Temperature-dependent ΔR/R of ML PTCDA. PTCDI and PTCDA reproduce all the main features of Me-PTCDI.

ω_{0-0}/eV	ω_{vib}/eV	λ^2	${\lambda_+}^2$	${\lambda_-}^2$
2.60	0.19	1.4	0.9	1.3
orientation		inter-layer	intra-layer(∥)	intra-layer(⊥)
Me-PTCDI (102) plane	J/meV	194.64	-28.85	1.36
Ref: E.Hadicke, F. Graser,	D_{e}/meV	136.56	0.63	9.55
Acta Cryst, **1986**, C42, 189.	D_{h}/meV	49.36	0.22	3.26
Molecular Mechanics Simulation	J/meV	-	-22.85	15.03
	D_{e}/meV	-	4.16	1.22
	D_{h}/meV	-	1.36	0.24

Supplementary Table 1. Hamiltonian parameters for different ML structures of Me-PTCDI derived from first principles DFT and TDDFT calculations.

	Superradiant state	Single molecule
ML sample on quartz	0.6848	0.1971
ML sample on SiO_2/Si	0.9141	0.1383
Multi-layer on SiO_2/Si	0.07195 (Frenkel state)	

Supplementary Table 2. Calculated oscillator strength based on transfer matrix modeling of differential reflectance of ML and multi-layer Me-PTCDI at 4K.

		inter-layer	intra-layer(∥)	intra-layer(∦)
Me-PTCDI (102) plane	electron	0.06	3056	13.21
	hole	0.09	4315	19.70
Molecular Mechanics Simulation	electron	-	69.55	805.8
	hole	-	112.8	3687

Supplementary Table 3. Calculated time scale (in unit of ps) for inter-molecular electron/hole charge transfer in Me-PTCDI crystals.

Supplementary References:

1. Hoffmann, M. et al. The lowest energy Frenkel and charge-transfer excitons in quasi-one-dimensional structures: application to MePTCDI and PTCDA crystals. *Chem. Phys.* **258**, 73-96, (2000).
2. Robert, C. et al. Optical spectroscopy of excited exciton states in MoS_2 monolayers in van der Waals heterostructures. *Phys. Rev. Materials* **2**, 011001(R) (2018).
3. Bahaa E. A. Saleh, BE. A. & Teich, M. C. *Fundamentals of Photonics*. (John Wiley & Sons, Inc., Press, USA, 1991).
4. Forrest, S. R. Ultrathin Organic films grown by organic molecular beam deposition and related techniques. *Chem. Rev*. **97**, 1793-1896 (1997).
